# Children’s IQs: Trasande et al. Respond

**Published:** 2006-07

**Authors:** Leonardo Trasande, Phillip J. Landrigan, Clyde B. Schechter

**Affiliations:** Center for Children’s Health and the Environment, Department of Community Medicine, Mount Sinai School of Medicine, New York, New York, E-mail: leo.trasande@mssm.edu; Department of Family Medicine, Albert Einstein College of Medicine, Bronx, New York

Schwartz makes a number of claims regarding our methodology (Trasande et al. 2005) that are inaccurate and based on a selective reading of the literature.

In our article (Trasande et al. 2005), we estimated the health and economic consequences of prenatal methylmercury (MeHg) exposure in the 2000 U.S. birth cohort. Our major findings were that at least 316,588 children in that birth cohort suffered IQ (intelligence quotient) loss of 0.2–24.4 points as a result of MeHg toxicity sustained *in utero*. This loss of intelligence causes diminished economic productivity that will persist, and this lost productivity is the major monetary consequence of methylmercury toxicity. We used the most up-to-date publicly available data on mercury exposures and health outcomes, applied a risk assessment approach developed by the National Research Council (NRC 1994), and made conservative assumptions throughout.

To compute decrements in IQ that resulted from prenatal mercury exposures, we used data from Mahaffey et al. (2004) on percentages of women of childbearing age in 1999–2000 with mercury concentrations ≥ 3.5, 4.84, 5.8, 7.13, and 15.0 μg/L. These data most closely reflect exposure to women in the years 1999–2000, when toxicity to the developing brains of children in the 2000 birth cohort would have occurred. We then applied logarithmic and linear models to these data, and we calculated a range of IQ decrements for each subpopulation born with a cord blood mercury concentration > 5.8 μg/L. To assess a range of possible outcomes, we conducted a sensitivity analysis in which we applied a range of IQ decrements for each increase in mercury concentration. We described our methods in great detail (Trasande et al. 2005). Through this series of calculations, we generated upper and lower ranges of possible IQ decrements for each subpopulation among the most highly exposed children in the 2000 U.S. birth cohort.

In his letter, Schwartz asserts that it is impossible to impute effects on children’s intelligence of prenatal exposures to mercury near the U.S. Environmental Protection Agency’s (EPA) reference dose (RfD). In proffering this assertion, he appears to ignore a recent meta-analysis of the three studies that confirmed a dose–response relationship between low-level prenatal MeHg exposure and IQ (Cohen et al. 2005). A recent U.S. cohort study has also detected decrements in visual recognition memory among children exposed prenatally to MeHg (Oken et al. 2005).

Schwartz suggests that we should have used the U.S. EPA benchmark dose level (BMDL) of 58 μg/L as a cutoff. He apparently assumes that no injury occurs to fetal brains from exposure to MeHg below that level. That approach does not reflect biologic or epidemiologic reality. We based our selection of 5.8 μg/L as a no adverse effect level on the epidemiologic evidence, not on the U.S. EPA’s regulatory documents (Budtz-Jorgensen et al. 2004; Grandjean et al. 1999; Kjellstrom et al. 1986 Kjellstrom et al. 1989). We relied especially upon the NRC’s report on prenatal exposure to MeHg (NRC 2000), which concluded that the likelihood of subnormal scores on neuro-developmental tests increased as cord blood mercury concentrations increased from levels as low as 5 μg/L. Methylmercury exposure has also been associated with persistent delays in peak I–III brainstem-evoked potentials at cord blood levels < 5 μg/L (Murata et al. 2004).

Schwartz misrepresents Crump et al.’s findings (1998), stating that they “superseded previous reports and found no IQ reduction.” In fact, the NRC (2000) stated that Crump et al.

reported nonsignificant results from a regression analysis on all the children in the New Zealand cohort, but [that these results became significant] after omission of a single child whose mother’s hair Hg concentration was 86 ppm (4 times higher than that of the next highest exposure level in the study).

Schwartz misrepresents our characterization of the Seychelles Islands study (Landrigan and Goldman 2003; Myers et al. 2003), accusing us of stating that it had half the statistical power of the Faroe Islands study (Grandjean et al. 1999). In actuality, we stated that the Seychelles study “had only 50% statistical power to detect the effects observed in the Faroes” (Trasande et al. 2005). Schwartz asserts that the NRC’s choice not to apply the Seychelles data in setting an RfD represents equivocation about the health effects of MeHg. In actuality, the NRC came to the same conclusion as we did: “[t]he weight of the evidence of developmental neurotoxic effects from exposure to MeHg is strong” (NRC 2000).

Recent work (Trasande et al. 2006) suggests that our calculation of the economic costs (Trasande et al. 2005) may, in fact, be an underestimate. The new study indicates that downward shifts in IQ are also associated with thousands of excess cases of mental retardation (defined as IQ < 70) in the United States each year. Care of these children is associated with needs for health care, special education, and other services that impose a great burden on society.

All of these adverse consequences can be prevented by prevention of prenatal exposure to MeHg.

## References

[b1-ehp0114-a00400] Budtz-Jorgensen EB Debes F Weihe P Grandjean P 2004. Adverse mercury effects in 7 year-old children expressed as loss in “IQ.” In: Recent Manuscripts. Available: http://www.chef-project.dk [accessed 1 April 2006].

[b2-ehp0114-a00400] Cohen JT, Bellinger DC, Shaywitz BA (2005). A quantitative analysis of prenatal methyl mercury exposure and cognitive
development. Am J Prev Med.

[b3-ehp0114-a00400] Crump KS, Kjellstrom T, Shipp AM, Silvers A, Stewart A (1998). Influence of prenatal mercury exposure upon scholastic and psychological
test performance: benchmark analysis of a New Zealand cohort. Risk Anal.

[b4-ehp0114-a00400] Grandjean P, Budtz-Jorgensen E, White RF, Jorgensen PJ, Weihe P, Debes F (1999). Methylmercury exposure biomarkers as indicators of neurotoxicity in children
aged 7 years. Am J Epidemiol.

[b5-ehp0114-a00400] Kjellstrom T Kennedy P Wallis S Mantell C 1986. Physical and Mental Development of Children with Prenatal Exposure to Mercury from Fish. Stage I. Preliminary Tests at Age 4. Report 3080. Solna, Sweden:National Swedish Environmental Protection Board.

[b6-ehp0114-a00400] Kjellstrom T Kennedy P Wallis S Stewart A Freiberg L Lind B 1986. Physical and Mental Development of Children with Prenatal Exposure to Mercury from Fish. Stage II. Interviews and Psychological Tests at Age 6. Report 3642. Solna, Sweden:National Swedish Environmental Protection Board.

[b7-ehp0114-a00400] Landrigan PJ, Goldman L (2003). Prenatal methylmercury exposure in the Seychelles [Letter]. Lancet.

[b8-ehp0114-a00400] Mahaffey KR, Clickner RP, Bodurow CC (2004). Blood organic mercury and dietary mercury intake: National Health and Nutrition
Examination Survey, 1999 and 2000. Environ Health Perspect.

[b9-ehp0114-a00400] Myers GJ, Davidson PW, Cox C, Shamlaye CF, Palumbo D, Cernichiari E (2003). Prenatal methylmercury exposure from ocean fish consumption in the Seychelles
child development study. Lancet.

[b10-ehp0114-a00400] Murata K, Weihe P, Budtz-Jorgensen E, Jorgensen PJ, Grandjean P (2004). Delayed brainstem auditory evoked potential latencies in 14-year-old children
exposed to methylmercury. J Pediatr.

[b11-ehp0114-a00400] Oken E, Wright RO, Kleinman KP, Bellinger D, Amarasiriwardena CJ, Hu H (2005). Maternal fish consumption, hair mercury, and infant cognition in a U.S. Cohort. Environ Health Perspect.

[b12-ehp0114-a00400] NRC (National Research Council) 1994. Science and Judgment in Risk Assessment. Washington, DC:National Academy Press.

[b13-ehp0114-a00400] NRC (National Research Council) 2000. Toxicological Effects of Methylmercury. Washington, DC:National Academy Press.

[b14-ehp0114-a00400] Trasande L, Landrigan PJ, Schechter C (2005). Public health and economic consequences of environmental methyl mercury
toxicity to the developing brain. Environ Health Perspect.

[b15-ehp0114-a00400] Trasande L, Schechter C, Haynes KA, Landrigan PJ (2006). Mental retardation and prenatal methylmercury toxicity. Am J Ind Med.

